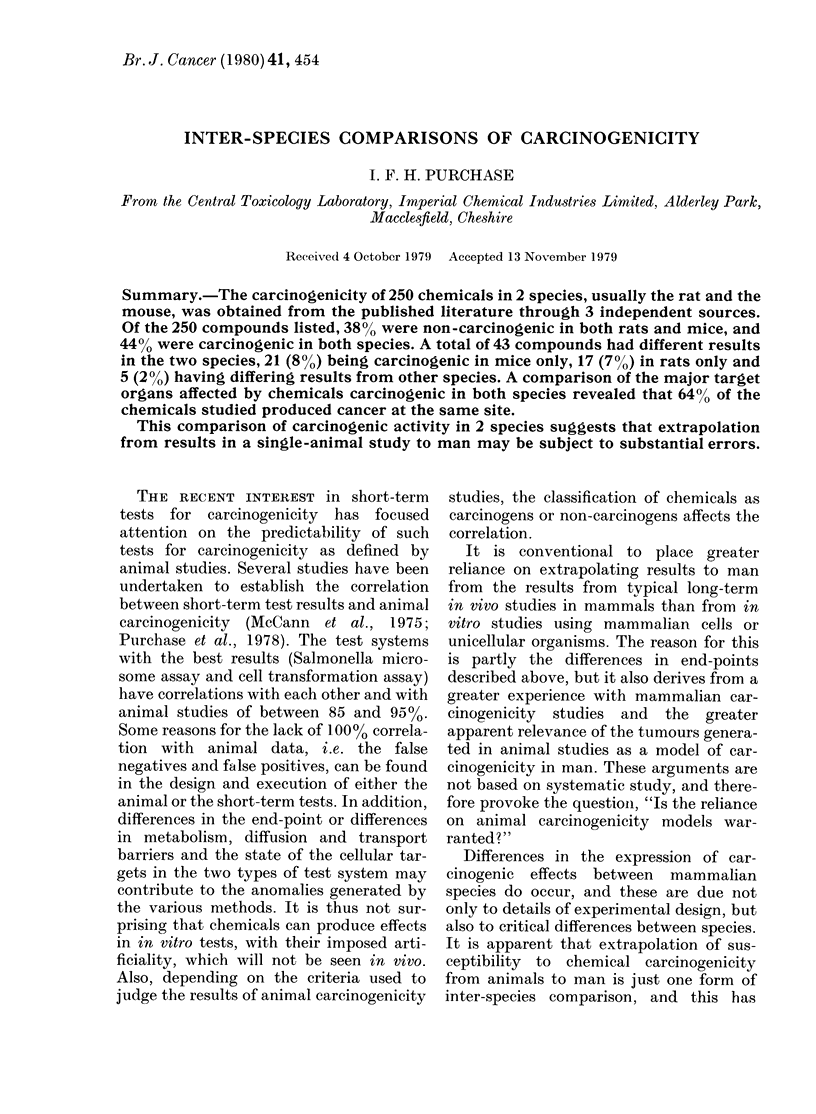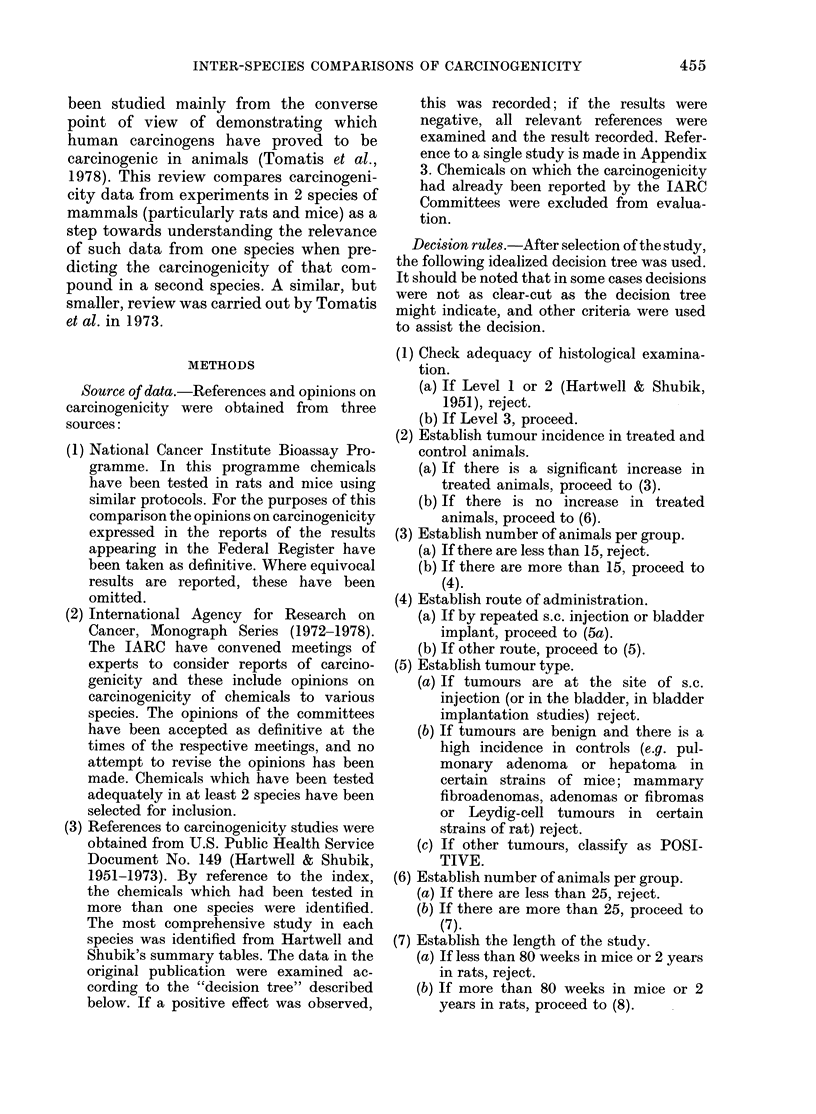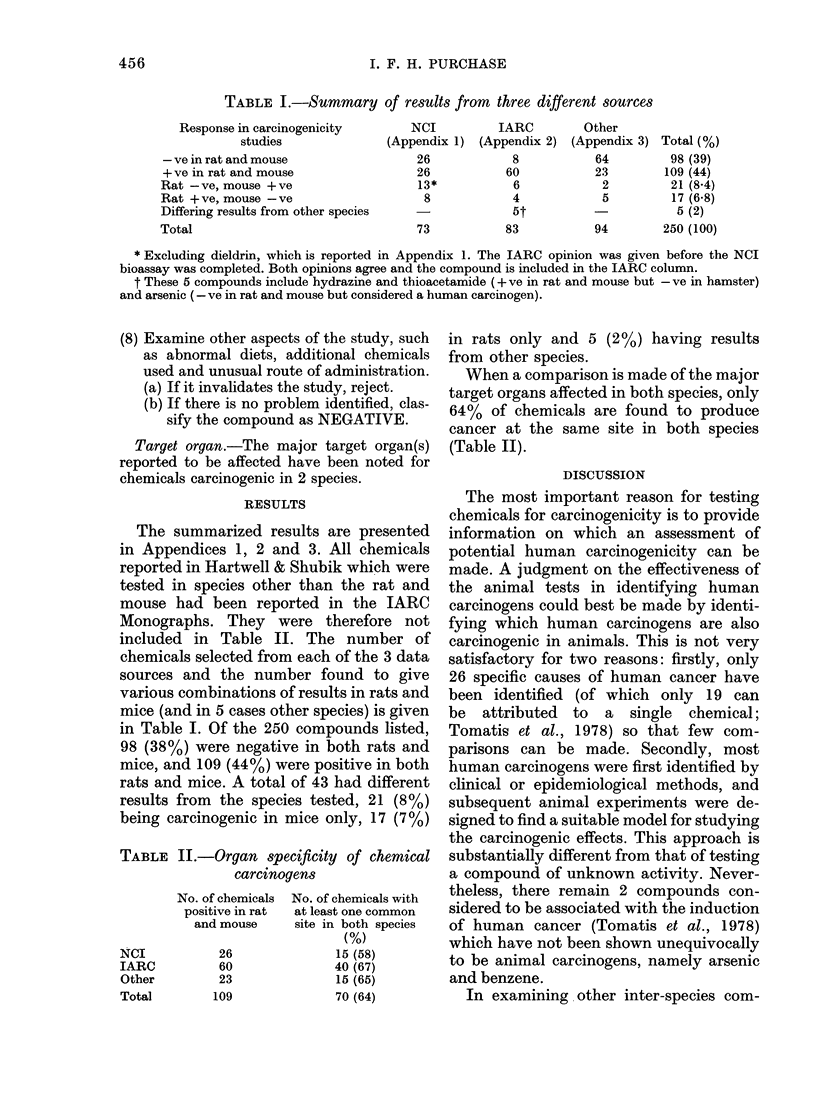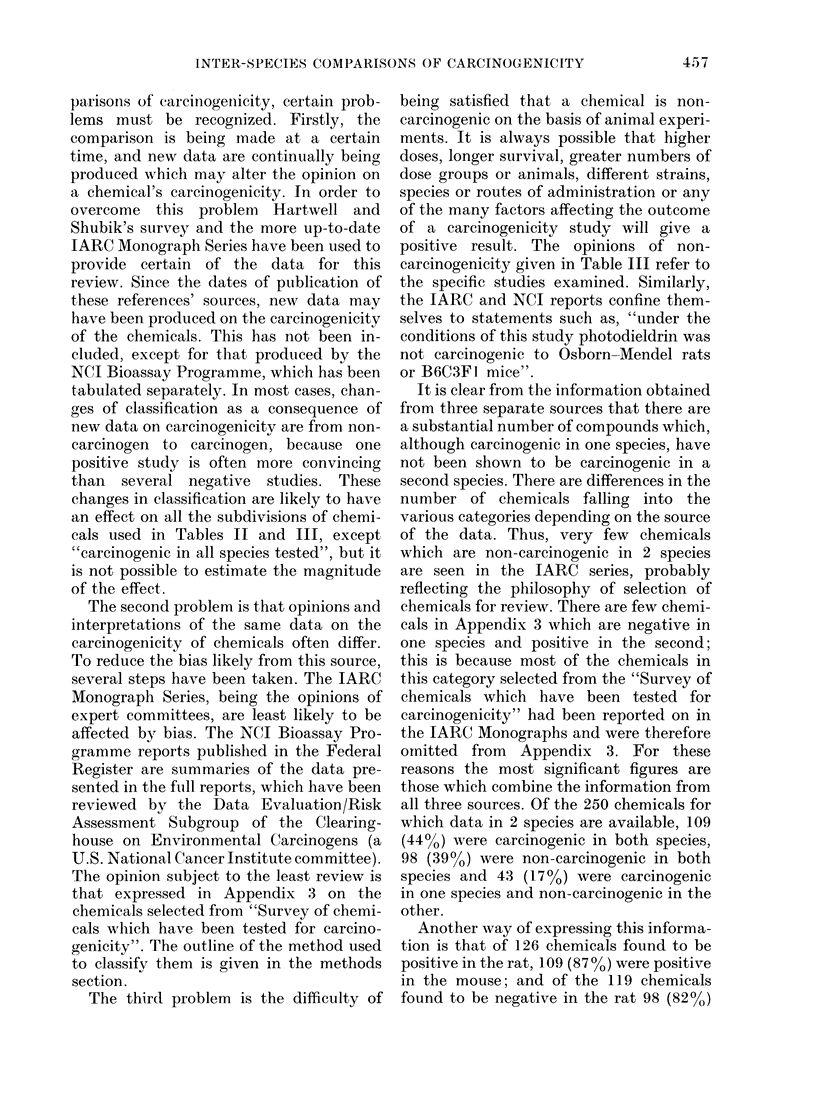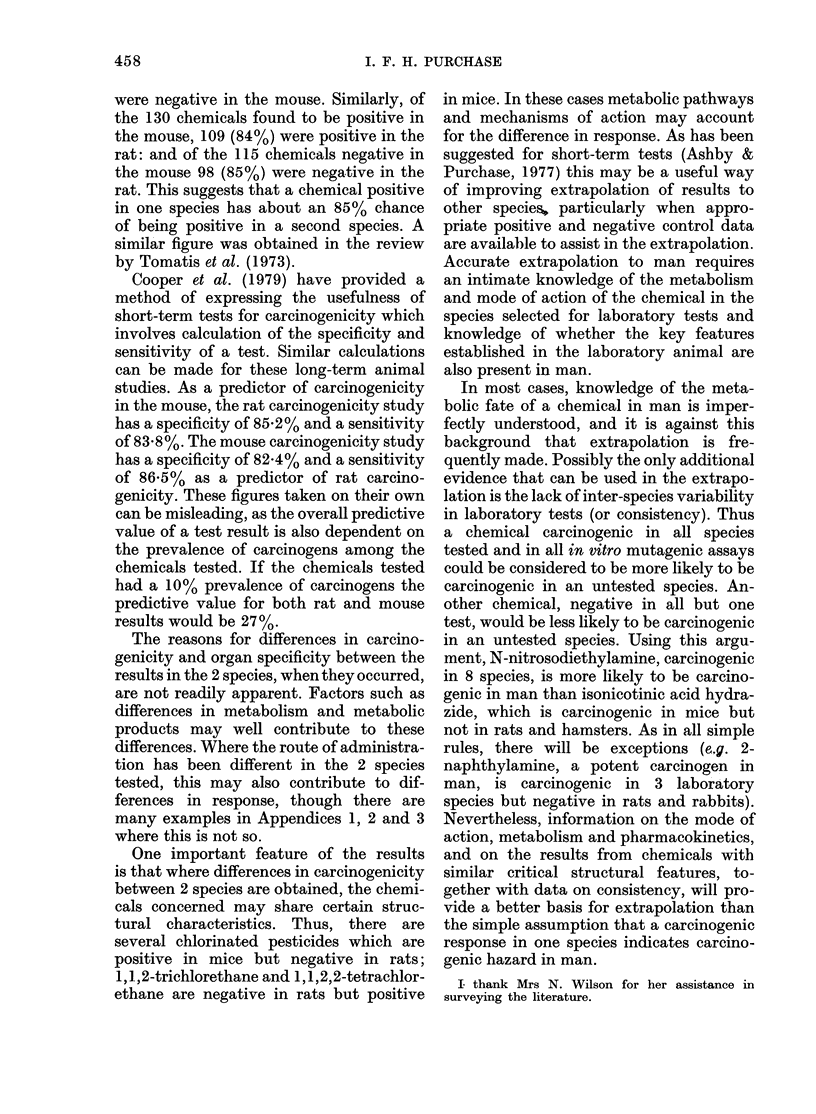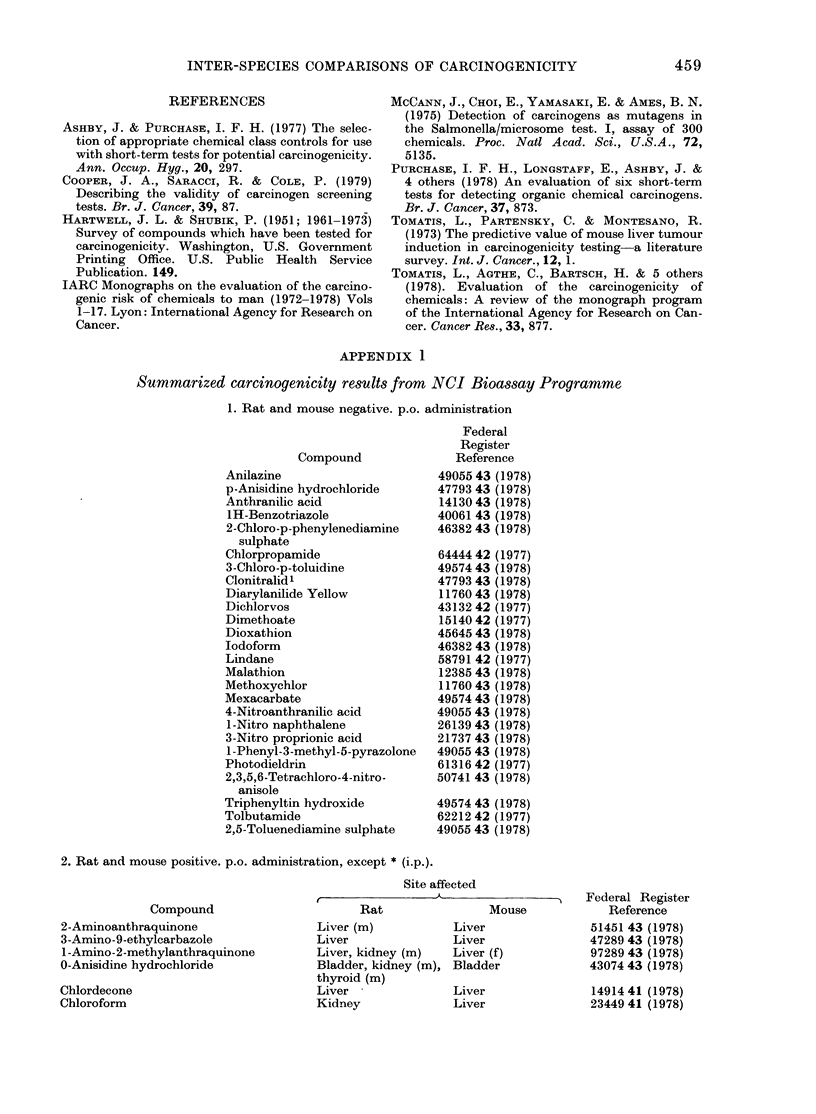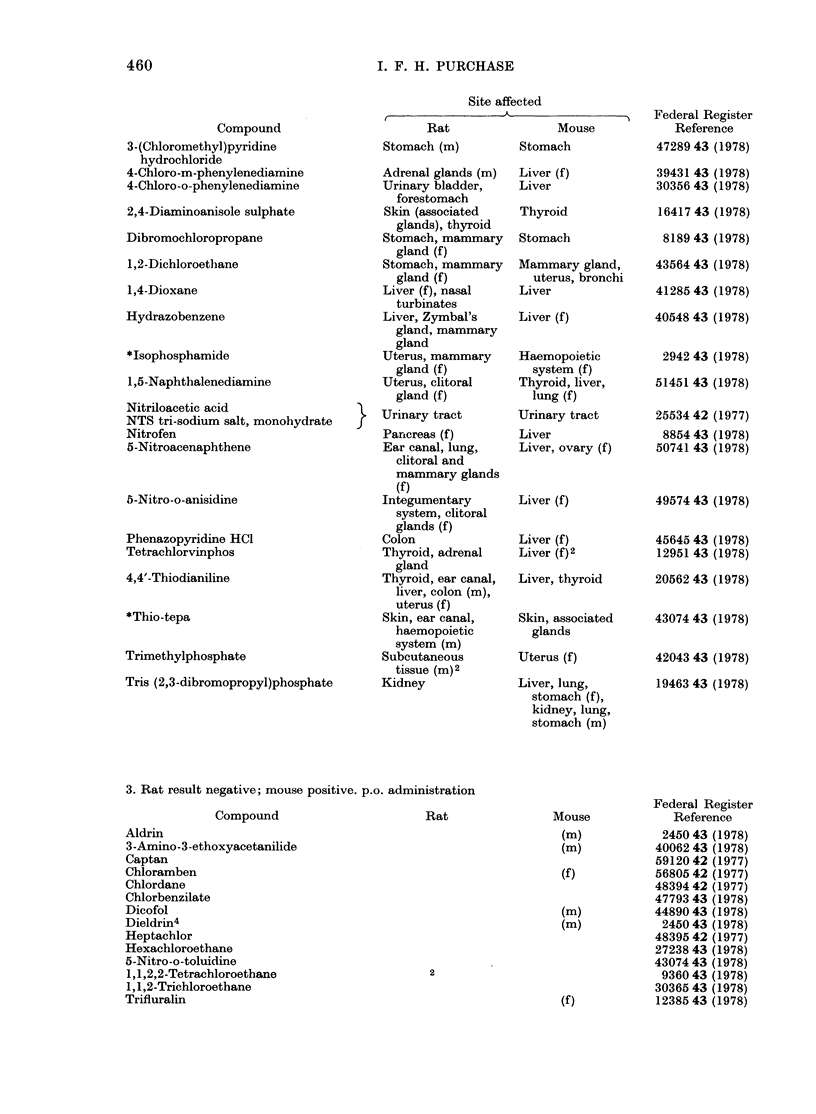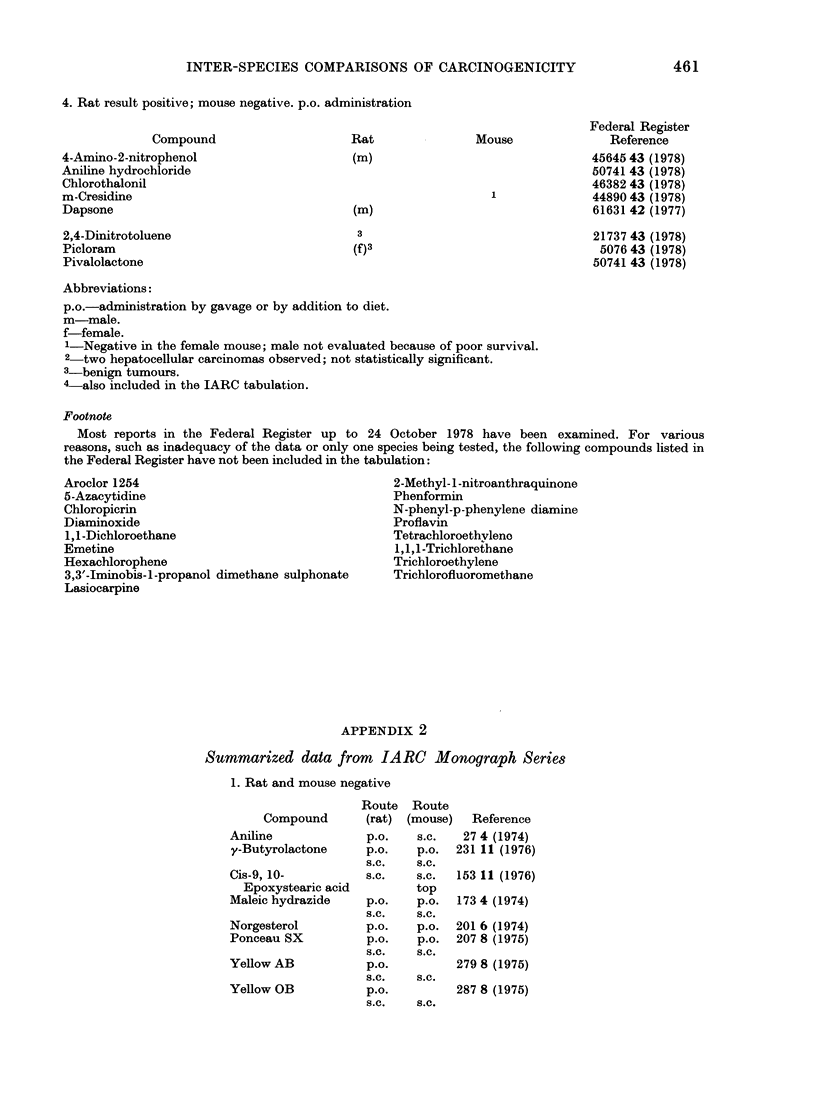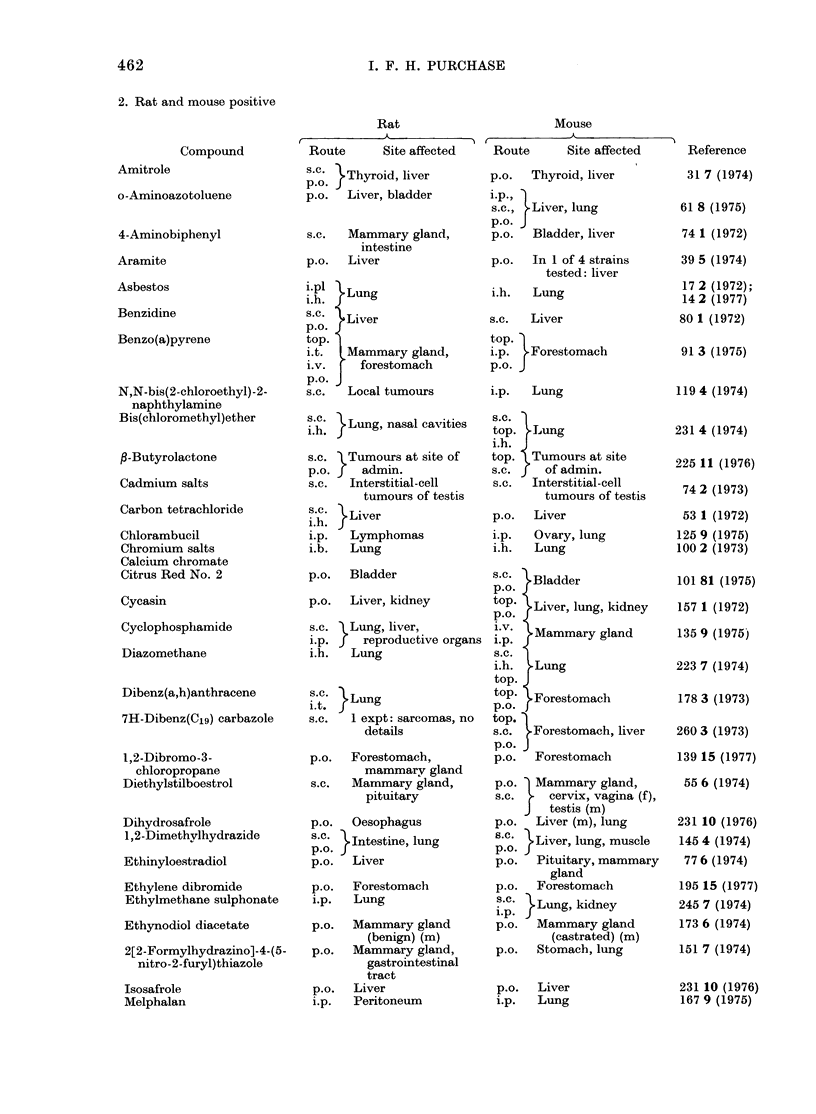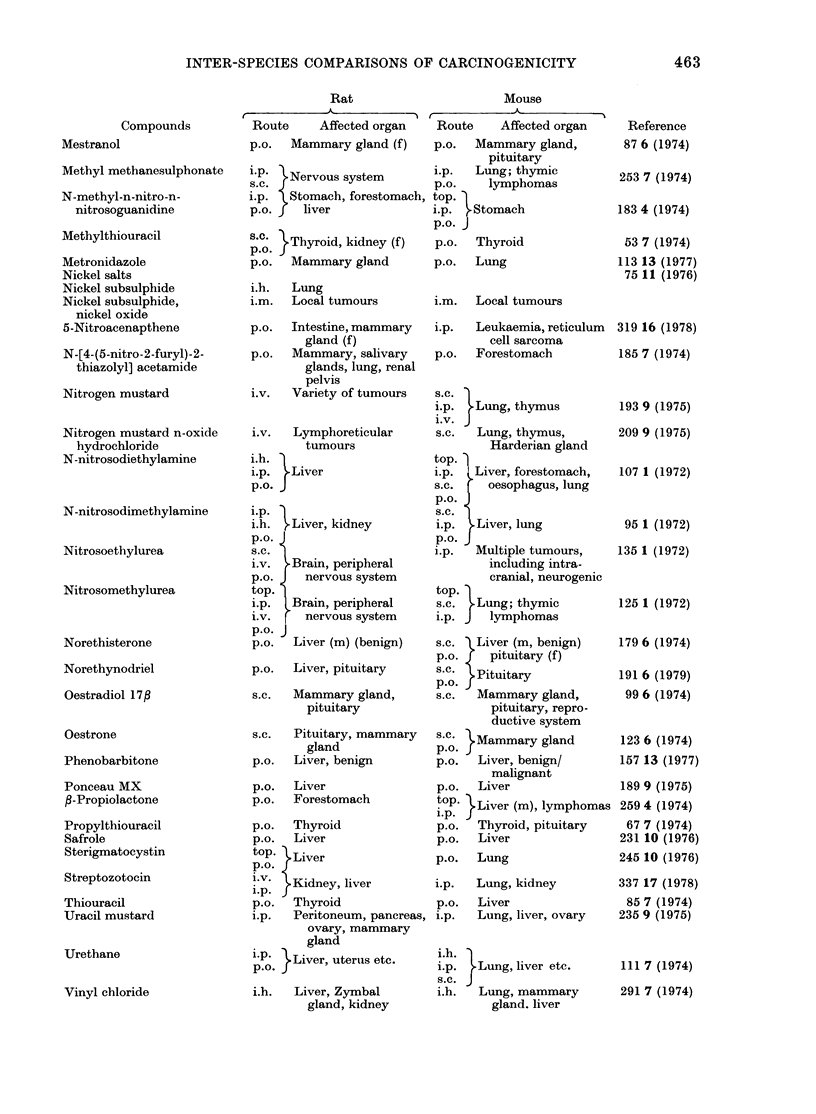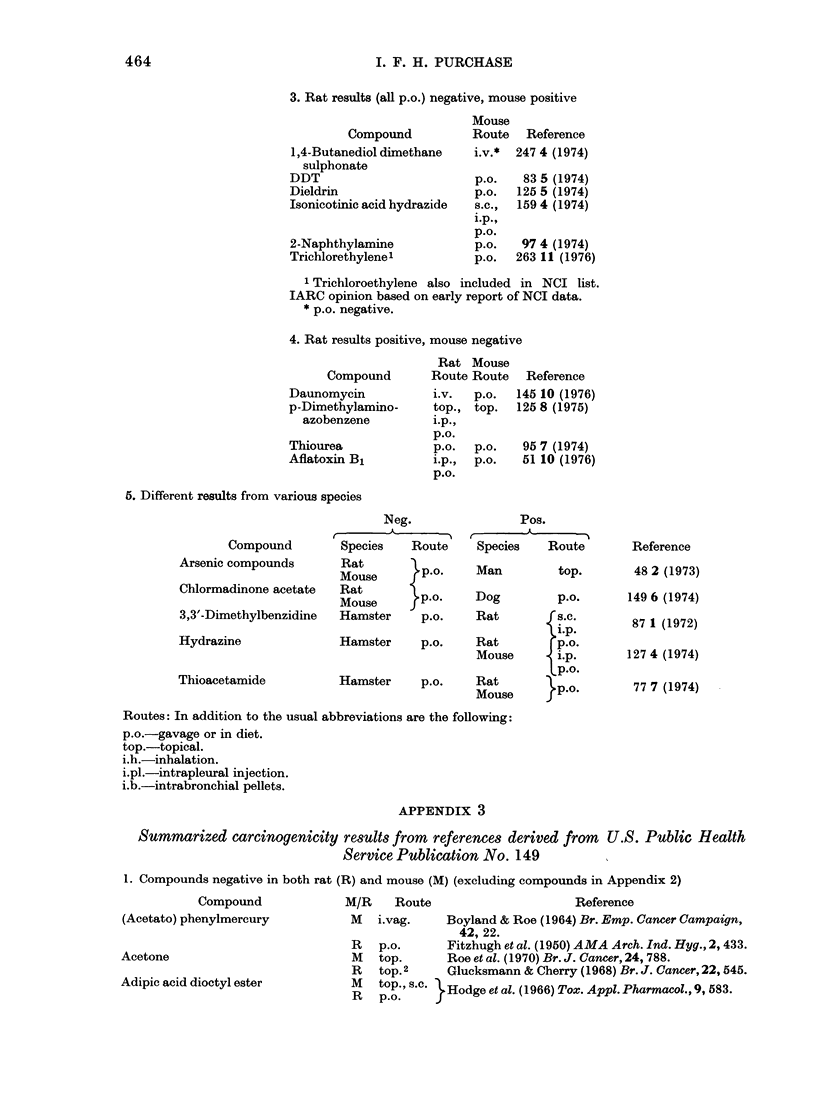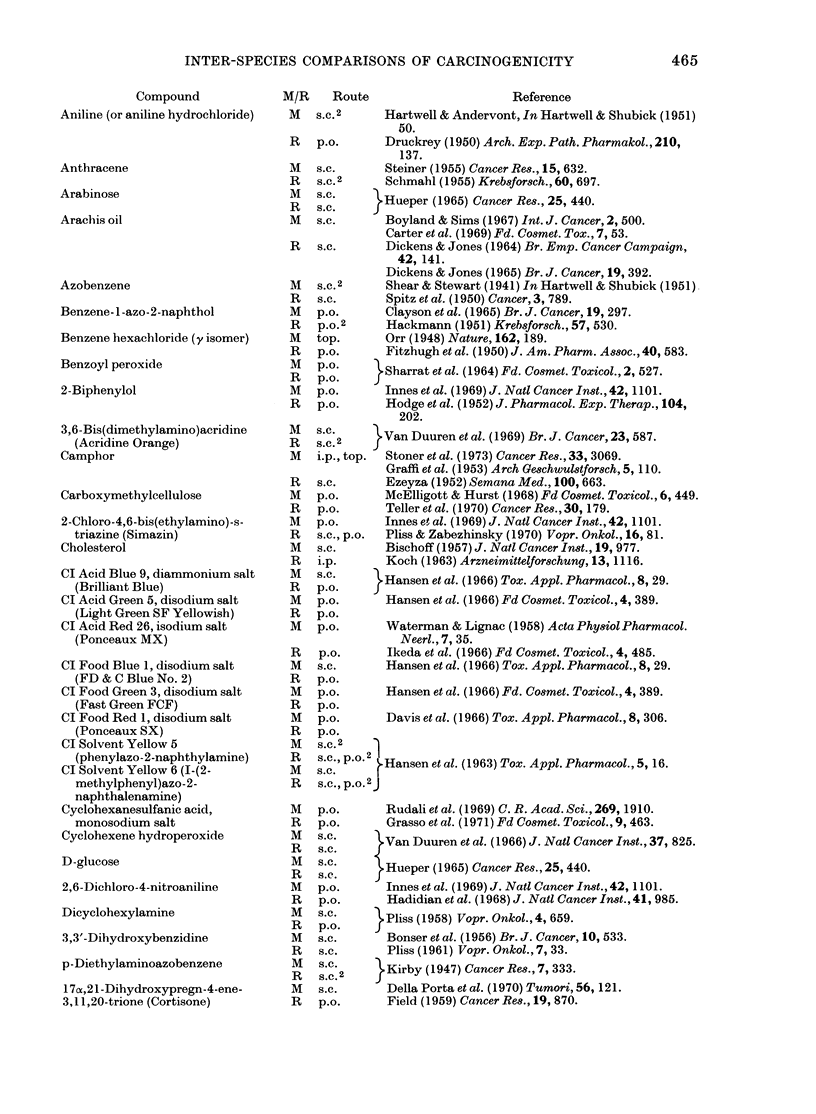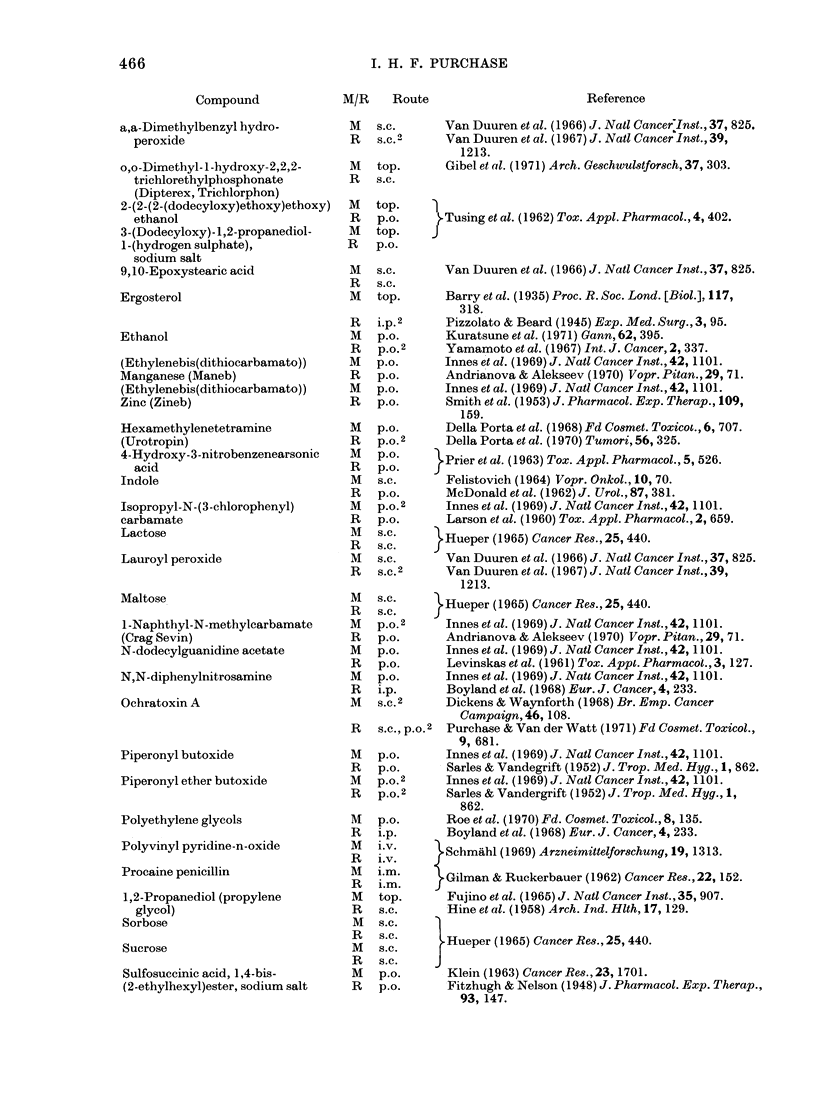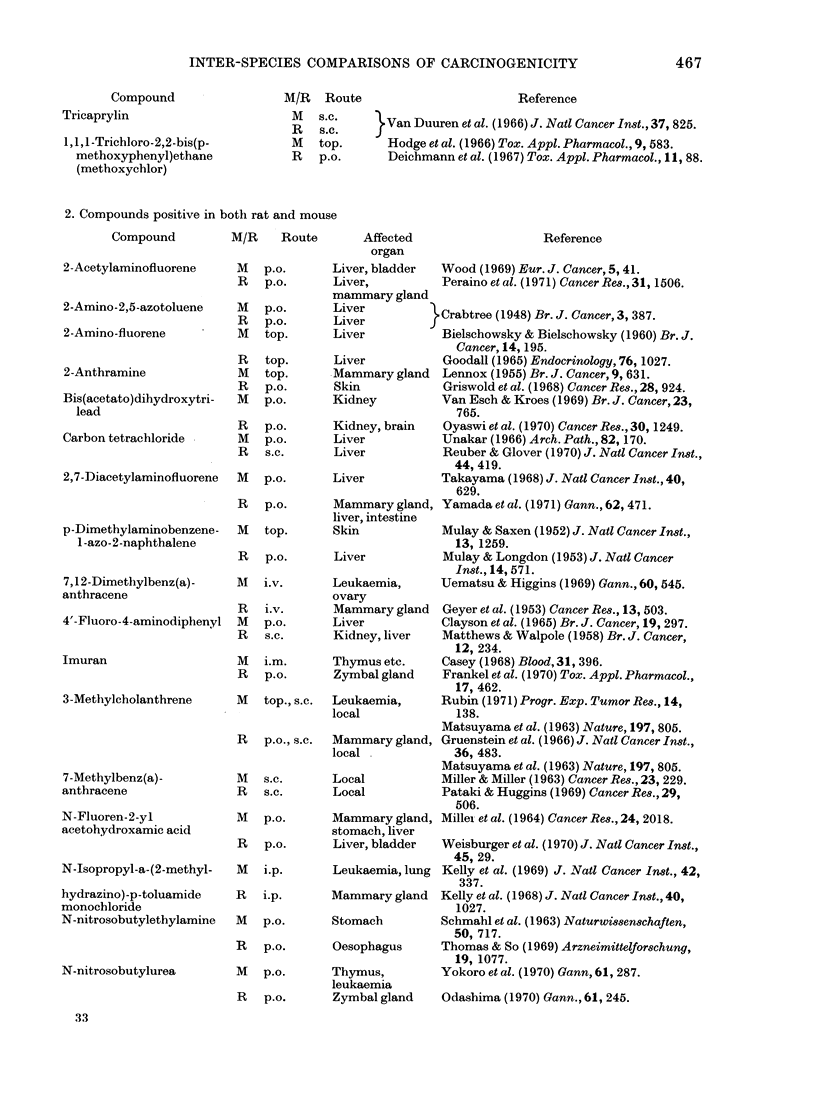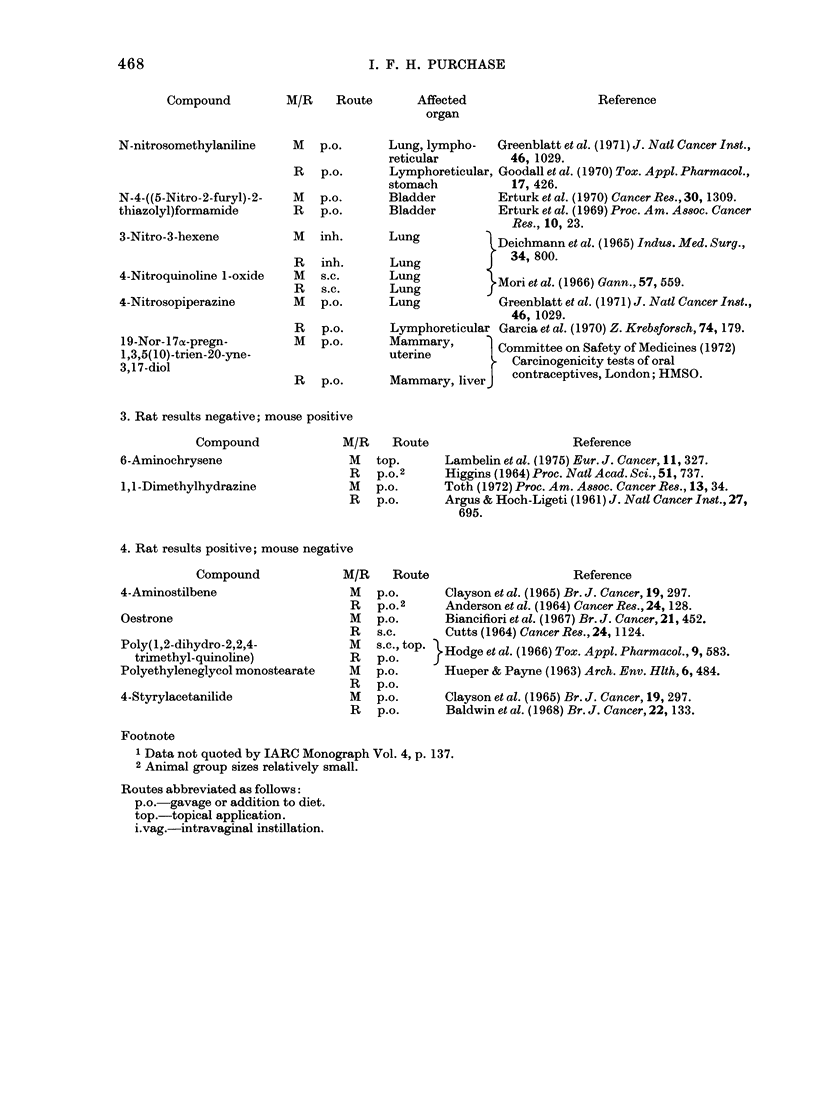# Inter-species comparisons of carcinogenicity.

**DOI:** 10.1038/bjc.1980.70

**Published:** 1980-03

**Authors:** I. F. Purchase

## Abstract

The carcinogenicity of 250 chemicals in 2 species, usually the rat and the mouse, was obtained from the published literature through 3 independent sources. Of the 250 compounds listed, 38% were non-carcinogenic in both rats and mice, and 44% were carcinogenic in both species. A total of 43 compounds had different results in the two species, 21 (8%) being carcinogenic in mice only, 17 (7%) in rats only and 5 (2%) having differing results from other species. A comparison of the major target organs affected by chemicals carcinogenic in both species revealed that 64% of the chemicals studied produced cancer at the same site. This comparison of carcinogenic activity in 2 species suggests that extrapolation from results in a single-animal study to man may be subject to substantial errors.


*Br. J. Cancer* (1980) **41**, 454

### INTER-SPECIES COMPARISONS OF CARCINOGENICITY

I. F. H. PURCHASE

*From the Central Toxicology Laboratory, Imperial Chemical Industries Limited, Alderley Park, Macclesfield, Cheshire*

Received 4 October 1979 Accepted 13 November 1979

**Summary.—The carcinogenicity of 250 chemicals in 2 species, usually the rat and the mouse, was obtained from the published literature through 3 independent sources. Of the 250 compounds listed, 38% were non-carcinogenic in both rats and mice, and 44% were carcinogenic in both species. A total of 43 compounds had different results in the two species, 21 (8%) being carcinogenic in mice only, 17 (7%) in rats only and 5 (2%) having differing results from other species. A comparison of the major target organs affected by chemicals carcinogenic in both species revealed that 64% of the chemicals studied produced cancer at the same site.**

**This comparison of carcinogenic activity in 2 species suggests that extrapolation from results in a single-animal study to man may be subject to substantial errors.**

THE RECENT INTEREST in short-term tests for carcinogenicity has focused attention on the predictability of such tests for carcinogenicity as defined by animal studies. Several studies have been undertaken to establish the correlation between short-term test results and animal carcinogenicity (McCann *et al.*, 1975; Purchase *et al.*, 1978). The test systems with the best results (Salmonella microsome assay and cell transformation assay) have correlations with each other and with animal studies of between 85 and 95%. Some reasons for the lack of 100% correlation with animal data, *i.e.* the false negatives and false positives, can be found in the design and execution of either the animal or the short-term tests. In addition, differences in the end-point or differences in metabolism, diffusion and transport barriers and the state of the cellular targets in the two types of test system may contribute to the anomalies generated by the various methods. It is thus not surprising that chemicals can produce effects in *in vitro* tests, with their imposed artificiality, which will not be seen *in vivo*. Also, depending on the criteria used to judge the results of animal carcinogenicity studies, the classification of chemicals as carcinogens or non-carcinogens affects the correlation.

It is conventional to place greater reliance on extrapolating results to man from the results from typical long-term *in vivo* studies in mammals than from *in vitro* studies using mammalian cells or unicellular organisms. The reason for this is partly the differences in end-points described above, but it also derives from a greater experience with mammalian carcinogenicity studies and the greater apparent relevance of the tumours generated in animal studies as a model of carcinogenicity in man. These arguments are not based on systematic study, and therefore provoke the question, "Is the reliance on animal carcinogenicity models warranted?"

Differences in the expression of carcinogenic effects between mammalian species do occur, and these are due not only to details of experimental design, but also to critical differences between species. It is apparent that extrapolation of susceptibility to chemical carcinogenicity from animals to man is just one form of inter-species comparison, and this has been studied mainly from the converse point of view of demonstrating which human carcinogens have proved to be carcinogenic in animals (Tomatis *et al.*, 1978). This review compares carcinogenicity data from experiments in 2 species of mammals (particularly rats and mice) as a step towards understanding the relevance of such data from one species when predicting the carcinogenicity of that compound in a second species. A similar, but smaller, review was carried out by Tomatis *et al.* in 1973.

### METHODS

*Source of data.*—References and opinions on carcinogenicity were obtained from three sources:

(1) National Cancer Institute Bioassay Programme. In this programme chemicals have been tested in rats and mice using similar protocols. For the purposes of this comparison the opinions on carcinogenicity expressed in the reports of the results appearing in the Federal Register have been taken as definitive. Where equivocal results are reported, these have been omitted.
(2) International Agency for Research on Cancer, Monograph Series (1972–1978). The IARC have convened meetings of experts to consider reports of carcinogenicity and these include opinions on carcinogenicity of chemicals to various species. The opinions of the committees have been accepted as definitive at the times of the respective meetings, and no attempt to revise the opinions has been made. Chemicals which have been tested adequately in at least 2 species have been selected for inclusion.
(3) References to carcinogenicity studies were obtained from U.S. Public Health Service Document No. 149 (Hartwell & Shubik, 1951–1973). By reference to the index, the chemicals which had been tested in more than one species were identified. The most comprehensive study in each species was identified from Hartwell and Shubik's summary tables. The data in the original publication were examined according to the "decision tree" described below. If a positive effect was observed, this was recorded; if the results were negative, all relevant references were examined and the result recorded. Reference to a single study is made in Appendix 3. Chemicals on which the carcinogenicity had already been reported by the IARC Committees were excluded from evaluation.

*Decision rules.*—After selection of the study, the following idealized decision tree was used. It should be noted that in some cases decisions were not as clear-cut as the decision tree might indicate, and other criteria were used to assist the decision.

(1) Check adequacy of histological examination.
    (a) If Level 1 or 2 (Hartwell & Shubik, 1951), reject.
    (b) If Level 3, proceed.
(2) Establish tumour incidence in treated and control animals.
    (a) If there is a significant increase in treated animals, proceed to (3).
    (b) If there is no increase in treated animals, proceed to (6).
(3) Establish number of animals per group.
    (a) If there are less than 15, reject.
    (b) If there are more than 15, proceed to (4).
(4) Establish route of administration.
    (a) If by repeated s.c. injection or bladder implant, proceed to (5*a*).
    (b) If other route, proceed to (5).
(5) Establish tumour type.
    (*a*) If tumours are at the site of s.c. injection (or in the bladder, in bladder implantation studies) reject.
    (*b*) If tumours are benign and there is a high incidence in controls (*e.g.* pulmonary adenoma or hepatoma in certain strains of mice; mammary fibroadenomas, adenomas or fibromas or Leydig-cell tumours in certain strains of rat) reject.
    (*c*) If other tumours, classify as POSITIVE.
(6) Establish number of animals per group.
    (*a*) If there are less than 25, reject.
    (*b*) If there are more than 25, proceed to (7).
(7) Establish the length of the study.
    (*a*) If less than 80 weeks in mice or 2 years in rats, reject.
    (*b*) If more than 80 weeks in mice or 2 years in rats, proceed to (8).

TABLE I.—*Summary of results from three different sources*

| Response in carcinogenicity studies | NCI (Appendix 1) | IARC (Appendix 2) | Other (Appendix 3) | Total (%) |
|---|---|---|---|---|
| −ve in rat and mouse | 26 | 8 | 64 | 98 (39) |
| +ve in rat and mouse | 26 | 60 | 23 | 109 (44) |
| Rat −ve, mouse +ve | 13* | 6 | 2 | 21 (8·4) |
| Rat +ve, mouse −ve | 8 | 4 | 5 | 17 (6·8) |
| Differing results from other species | — | 5† | — | 5 (2) |
| Total | 73 | 83 | 94 | 250 (100) |

* Excluding dieldrin, which is reported in Appendix 1. The IARC opinion was given before the NCI bioassay was completed. Both opinions agree and the compound is included in the IARC column.

† These 5 compounds include hydrazine and thioacetamide (+ve in rat and mouse but −ve in hamster) and arsenic (−ve in rat and mouse but considered a human carcinogen).

(8) Examine other aspects of the study, such as abnormal diets, additional chemicals used and unusual route of administration.
  (a) If it invalidates the study, reject.
  (b) If there is no problem identified, classify the compound as NEGATIVE.

*Target organ.*—The major target organ(s) reported to be affected have been noted for chemicals carcinogenic in 2 species.

### RESULTS

The summarized results are presented in Appendices 1, 2 and 3. All chemicals reported in Hartwell & Shubik which were tested in species other than the rat and mouse had been reported in the IARC Monographs. They were therefore not included in Table II. The number of chemicals selected from each of the 3 data sources and the number found to give various combinations of results in rats and mice (and in 5 cases other species) is given in Table I. Of the 250 compounds listed, 98 (38%) were negative in both rats and mice, and 109 (44%) were positive in both rats and mice. A total of 43 had different results from the species tested, 21 (8%) being carcinogenic in mice only, 17 (7%) in rats only and 5 (2%) having results from other species.

TABLE II.—*Organ specificity of chemical carcinogens*

| | No. of chemicals positive in rat and mouse | No. of chemicals with at least one common site in both species (%) |
|---|---|---|
| NCI | 26 | 15 (58) |
| IARC | 60 | 40 (67) |
| Other | 23 | 15 (65) |
| Total | 109 | 70 (64) |

When a comparison is made of the major target organs affected in both species, only 64% of chemicals are found to produce cancer at the same site in both species (Table II).

### DISCUSSION

The most important reason for testing chemicals for carcinogenicity is to provide information on which an assessment of potential human carcinogenicity can be made. A judgment on the effectiveness of the animal tests in identifying human carcinogens could best be made by identifying which human carcinogens are also carcinogenic in animals. This is not very satisfactory for two reasons: firstly, only 26 specific causes of human cancer have been identified (of which only 19 can be attributed to a single chemical; Tomatis *et al.*, 1978) so that few comparisons can be made. Secondly, most human carcinogens were first identified by clinical or epidemiological methods, and subsequent animal experiments were designed to find a suitable model for studying the carcinogenic effects. This approach is substantially different from that of testing a compound of unknown activity. Nevertheless, there remain 2 compounds considered to be associated with the induction of human cancer (Tomatis *et al.*, 1978) which have not been shown unequivocally to be animal carcinogens, namely arsenic and benzene.

In examining other inter-species comparisons of carcinogenicity, certain problems must be recognized. Firstly, the comparison is being made at a certain time, and new data are continually being produced which may alter the opinion on a chemical's carcinogenicity. In order to overcome this problem Hartwell and Shubik's survey and the more up-to-date IARC Monograph Series have been used to provide certain of the data for this review. Since the dates of publication of these references' sources, new data may have been produced on the carcinogenicity of the chemicals. This has not been included, except for that produced by the NCI Bioassay Programme, which has been tabulated separately. In most cases, changes of classification as a consequence of new data on carcinogenicity are from non-carcinogen to carcinogen, because one positive study is often more convincing than several negative studies. These changes in classification are likely to have an effect on all the subdivisions of chemicals used in Tables II and III, except "carcinogenic in all species tested", but it is not possible to estimate the magnitude of the effect.

The second problem is that opinions and interpretations of the same data on the carcinogenicity of chemicals often differ. To reduce the bias likely from this source, several steps have been taken. The IARC Monograph Series, being the opinions of expert committees, are least likely to be affected by bias. The NCI Bioassay Programme reports published in the Federal Register are summaries of the data presented in the full reports, which have been reviewed by the Data Evaluation/Risk Assessment Subgroup of the Clearinghouse on Environmental Carcinogens (a U.S. National Cancer Institute committee). The opinion subject to the least review is that expressed in Appendix 3 on the chemicals selected from "Survey of chemicals which have been tested for carcinogenicity". The outline of the method used to classify them is given in the methods section.

The third problem is the difficulty of being satisfied that a chemical is non-carcinogenic on the basis of animal experiments. It is always possible that higher doses, longer survival, greater numbers of dose groups or animals, different strains, species or routes of administration or any of the many factors affecting the outcome of a carcinogenicity study will give a positive result. The opinions of non-carcinogenicity given in Table III refer to the specific studies examined. Similarly, the IARC and NCI reports confine themselves to statements such as, "under the conditions of this study photodieldrin was not carcinogenic to Osborn–Mendel rats or B6C3F1 mice".

It is clear from the information obtained from three separate sources that there are a substantial number of compounds which, although carcinogenic in one species, have not been shown to be carcinogenic in a second species. There are differences in the number of chemicals falling into the various categories depending on the source of the data. Thus, very few chemicals which are non-carcinogenic in 2 species are seen in the IARC series, probably reflecting the philosophy of selection of chemicals for review. There are few chemicals in Appendix 3 which are negative in one species and positive in the second; this is because most of the chemicals in this category selected from the "Survey of chemicals which have been tested for carcinogenicity" had been reported on in the IARC Monographs and were therefore omitted from Appendix 3. For these reasons the most significant figures are those which combine the information from all three sources. Of the 250 chemicals for which data in 2 species are available, 109 (44%) were carcinogenic in both species, 98 (39%) were non-carcinogenic in both species and 43 (17%) were carcinogenic in one species and non-carcinogenic in the other.

Another way of expressing this information is that of 126 chemicals found to be positive in the rat, 109 (87%) were positive in the mouse; and of the 119 chemicals found to be negative in the rat 98 (82%)

were negative in the mouse. Similarly, of the 130 chemicals found to be positive in the mouse, 109 (84%) were positive in the rat: and of the 115 chemicals negative in the mouse 98 (85%) were negative in the rat. This suggests that a chemical positive in one species has about an 85% chance of being positive in a second species. A similar figure was obtained in the review by Tomatis *et al.* (1973).

Cooper *et al.* (1979) have provided a method of expressing the usefulness of short-term tests for carcinogenicity which involves calculation of the specificity and sensitivity of a test. Similar calculations can be made for these long-term animal studies. As a predictor of carcinogenicity in the mouse, the rat carcinogenicity study has a specificity of 85·2% and a sensitivity of 83·8%. The mouse carcinogenicity study has a specificity of 82·4% and a sensitivity of 86·5% as a predictor of rat carcinogenicity. These figures taken on their own can be misleading, as the overall predictive value of a test result is also dependent on the prevalence of carcinogens among the chemicals tested. If the chemicals tested had a 10% prevalence of carcinogens the predictive value for both rat and mouse results would be 27%.

The reasons for differences in carcinogenicity and organ specificity between the results in the 2 species, when they occurred, are not readily apparent. Factors such as differences in metabolism and metabolic products may well contribute to these differences. Where the route of administration has been different in the 2 species tested, this may also contribute to differences in response, though there are many examples in Appendices 1, 2 and 3 where this is not so.

One important feature of the results is that where differences in carcinogenicity between 2 species are obtained, the chemicals concerned may share certain structural characteristics. Thus, there are several chlorinated pesticides which are positive in mice but negative in rats; 1,1,2-trichlorethane and 1,1,2,2-tetrachlorethane are negative in rats but positive in mice. In these cases metabolic pathways and mechanisms of action may account for the difference in response. As has been suggested for short-term tests (Ashby & Purchase, 1977) this may be a useful way of improving extrapolation of results to other species, particularly when appropriate positive and negative control data are available to assist in the extrapolation. Accurate extrapolation to man requires an intimate knowledge of the metabolism and mode of action of the chemical in the species selected for laboratory tests and knowledge of whether the key features established in the laboratory animal are also present in man.

In most cases, knowledge of the metabolic fate of a chemical in man is imperfectly understood, and it is against this background that extrapolation is frequently made. Possibly the only additional evidence that can be used in the extrapolation is the lack of inter-species variability in laboratory tests (or consistency). Thus a chemical carcinogenic in all species tested and in all *in vitro* mutagenic assays could be considered to be more likely to be carcinogenic in an untested species. Another chemical, negative in all but one test, would be less likely to be carcinogenic in an untested species. Using this argument, N-nitrosodiethylamine, carcinogenic in 8 species, is more likely to be carcinogenic in man than isonicotinic acid hydrazide, which is carcinogenic in mice but not in rats and hamsters. As in all simple rules, there will be exceptions (*e.g.* 2-naphthylamine, a potent carcinogen in man, is carcinogenic in 3 laboratory species but negative in rats and rabbits). Nevertheless, information on the mode of action, metabolism and pharmacokinetics, and on the results from chemicals with similar critical structural features, together with data on consistency, will provide a better basis for extrapolation than the simple assumption that a carcinogenic response in one species indicates carcinogenic hazard in man.

I thank Mrs N. Wilson for her assistance in surveying the literature.

### REFERENCES

ASHBY, J. & PURCHASE, I. F. H. (1977) The selection of appropriate chemical class controls for use with short-term tests for potential carcinogenicity. *Ann. Occup. Hyg.*, **20**, 297.

COOPER, J. A., SARACCI, R. & COLE, P. (1979) Describing the validity of carcinogen screening tests. *Br. J. Cancer*, **39**, 87.

HARTWELL, J. L. & SHUBIK, P. (1951; 1961–1973) Survey of compounds which have been tested for carcinogenicity. Washington, U.S. Government Printing Office. U.S. Public Health Service Publication. **149.**

IARC Monographs on the evaluation of the carcinogenic risk of chemicals to man (1972–1978) Vols 1–17. Lyon: International Agency for Research on Cancer.

MCCANN, J., CHOI, E., YAMASAKI, E. & AMES, B. N. (1975) Detection of carcinogens as mutagens in the Salmonella/microsome test. I, assay of 300 chemicals. *Proc. Natl Acad. Sci., U.S.A.*, **72**, 5135.

PURCHASE, I. F. H., LONGSTAFF, E., ASHBY, J. & 4 others (1978) An evaluation of six short-term tests for detecting organic chemical carcinogens. *Br. J. Cancer*, **37**, 873.

TOMATIS, L., PARTENSKY, C. & MONTESANO, R. (1973) The predictive value of mouse liver tumour induction in carcinogenicity testing—a literature survey. *Int. J. Cancer.*, **12**, 1.

TOMATIS, L., AGTHE, C., BARTSCH, H. & 5 others (1978). Evaluation of the carcinogenicity of chemicals: A review of the monograph program of the International Agency for Research on Cancer. *Cancer Res.*, **33**, 877.

### APPENDIX 1

### *Summarized carcinogenicity results from NCI Bioassay Programme*

1. Rat and mouse negative. p.o. administration

| Compound | Federal Register Reference |
|---|---|
| Anilazine | 49055 **43** (1978) |
| p-Anisidine hydrochloride | 47793 **43** (1978) |
| Anthranilic acid | 14130 **43** (1978) |
| 1H-Benzotriazole | 40061 **43** (1978) |
| 2-Chloro-p-phenylenediamine sulphate | 46382 **43** (1978) |
| Chlorpropamide | 64444 **42** (1977) |
| 3-Chloro-p-toluidine | 49574 **43** (1978) |
| Clonitralid[1] | 47793 **43** (1978) |
| Diarylanilide Yellow | 11760 **43** (1978) |
| Dichlorvos | 43132 **42** (1977) |
| Dimethoate | 15140 **42** (1977) |
| Dioxathion | 45645 **43** (1978) |
| Iodoform | 46382 **43** (1978) |
| Lindane | 58791 **42** (1977) |
| Malathion | 12385 **43** (1978) |
| Methoxychlor | 11760 **43** (1978) |
| Mexacarbate | 49574 **43** (1978) |
| 4-Nitroanthranilic acid | 49055 **43** (1978) |
| 1-Nitro naphthalene | 26139 **43** (1978) |
| 3-Nitro proprionic acid | 21737 **43** (1978) |
| 1-Phenyl-3-methyl-5-pyrazolone | 49055 **43** (1978) |
| Photodieldrin | 61316 **42** (1977) |
| 2,3,5,6-Tetrachloro-4-nitro-anisole | 50741 **43** (1978) |
| Triphenyltin hydroxide | 49574 **43** (1978) |
| Tolbutamide | 62212 **42** (1977) |
| 2,5-Toluenediamine sulphate | 49055 **43** (1978) |

2. Rat and mouse positive. p.o. administration, except * (i.p.).

| Compound | Site affected: Rat | Site affected: Mouse | Federal Register Reference |
|---|---|---|---|
| 2-Aminoanthraquinone | Liver (m) | Liver | 51451 **43** (1978) |
| 3-Amino-9-ethylcarbazole | Liver | Liver | 47289 **43** (1978) |
| 1-Amino-2-methylanthraquinone | Liver, kidney (m) | Liver (f) | 97289 **43** (1978) |
| 0-Anisidine hydrochloride | Bladder, kidney (m), thyroid (m) | Bladder | 43074 **43** (1978) |
| Chlordecone | Liver | Liver | 14914 **41** (1978) |
| Chloroform | Kidney | Liver | 23449 **41** (1978) |

| Compound | Site affected: Rat | Site affected: Mouse | Federal Register Reference |
|---|---|---|---|
| 3-(Chloromethyl)pyridine hydrochloride | Stomach (m) | Stomach | 47289 **43** (1978) |
| 4-Chloro-m-phenylenediamine | Adrenal glands (m) | Liver (f) | 39431 **43** (1978) |
| 4-Chloro-o-phenylenediamine | Urinary bladder, forestomach | Liver | 30356 **43** (1978) |
| 2,4-Diaminoanisole sulphate | Skin (associated glands), thyroid | Thyroid | 16417 **43** (1978) |
| Dibromochloropropane | Stomach, mammary gland (f) | Stomach | 8189 **43** (1978) |
| 1,2-Dichloroethane | Stomach, mammary gland (f) | Mammary gland, uterus, bronchi | 43564 **43** (1978) |
| 1,4-Dioxane | Liver (f), nasal turbinates | Liver | 41285 **43** (1978) |
| Hydrazobenzene | Liver, Zymbal's gland, mammary gland | Liver (f) | 40548 **43** (1978) |
| *Isophosphamide | Uterus, mammary gland (f) | Haemopoietic system (f) | 2942 **43** (1978) |
| 1,5-Naphthalenediamine | Uterus, clitoral gland (f) | Thyroid, liver, lung (f) | 51451 **43** (1978) |
| Nitriloacetic acid; NTS tri-sodium salt, monohydrate | Urinary tract | Urinary tract | 25534 **42** (1977) |
| Nitrofen | Pancreas (f) | Liver | 8854 **43** (1978) |
| 5-Nitroacenaphthene | Ear canal, lung, clitoral and mammary glands (f) | Liver, ovary (f) | 50741 **43** (1978) |
| 5-Nitro-o-anisidine | Integumentary system, clitoral glands (f) | Liver (f) | 49574 **43** (1978) |
| Phenazopyridine HCl | Colon | Liver (f) | 45645 **43** (1978) |
| Tetrachlorvinphos | Thyroid, adrenal gland | Liver (f)[2] | 12951 **43** (1978) |
| 4,4′-Thiodianiline | Thyroid, ear canal, liver, colon (m), uterus (f) | Liver, thyroid | 20562 **43** (1978) |
| *Thio-tepa | Skin, ear canal, haemopoietic system (m) | Skin, associated glands | 43074 **43** (1978) |
| Trimethylphosphate | Subcutaneous tissue (m)[2] | Uterus (f) | 42043 **43** (1978) |
| Tris (2,3-dibromopropyl)phosphate | Kidney | Liver, lung, stomach (f), kidney, lung, stomach (m) | 19463 **43** (1978) |

3. Rat result negative; mouse positive. p.o. administration

| Compound | Rat | Mouse | Federal Register Reference |
|---|---|---|---|
| Aldrin | | (m) | 2450 **43** (1978) |
| 3-Amino-3-ethoxyacetanilide | | (m) | 40062 **43** (1978) |
| Captan | | | 59120 **42** (1977) |
| Chloramben | | (f) | 56805 **42** (1977) |
| Chlordane | | | 48394 **42** (1977) |
| Chlorbenzilate | | | 47793 **43** (1978) |
| Dicofol | | (m) | 44890 **43** (1978) |
| Dieldrin[4] | | (m) | 2450 **43** (1978) |
| Heptachlor | | | 48395 **42** (1977) |
| Hexachloroethane | | | 27238 **43** (1978) |
| 5-Nitro-o-toluidine | | | 43074 **43** (1978) |
| 1,1,2,2-Tetrachloroethane | 2 | | 9360 **43** (1978) |
| 1,1,2-Trichloroethane | | | 30365 **43** (1978) |
| Trifluralin | | (f) | 12385 **43** (1978) |

4. Rat result positive; mouse negative. p.o. administration

| Compound | Rat | Mouse | Federal Register Reference |
|---|---|---|---|
| 4-Amino-2-nitrophenol | (m) | | 45645 **43** (1978) |
| Aniline hydrochloride | | | 50741 **43** (1978) |
| Chlorothalonil | | | 46382 **43** (1978) |
| m-Cresidine | | 1 | 44890 **43** (1978) |
| Dapsone | (m) | | 61631 **42** (1977) |
| 2,4-Dinitrotoluene | 3 | | 21737 **43** (1978) |
| Picloram | (f)3 | | 5076 **43** (1978) |
| Pivalolactone | | | 50741 **43** (1978) |

Abbreviations:

p.o.—administration by gavage or by addition to diet.
m—male.
f—female.
1—Negative in the female mouse; male not evaluated because of poor survival.
2—two hepatocellular carcinomas observed; not statistically significant.
3—benign tumours.
4—also included in the IARC tabulation.

*Footnote*

Most reports in the Federal Register up to 24 October 1978 have been examined. For various reasons, such as inadequacy of the data or only one species being tested, the following compounds listed in the Federal Register have not been included in the tabulation:

Aroclor 1254
5-Azacytidine
Chloropicrin
Diaminoxide
1,1-Dichloroethane
Emetine
Hexachlorophene
3,3′-Iminobis-1-propanol dimethane sulphonate
Lasiocarpine
2-Methyl-1-nitroanthraquinone
Phenformin
N-phenyl-p-phenylene diamine
Proflavin
Tetrachloroethyleno
1,1,1-Trichlorethane
Trichloroethylene
Trichlorofluoromethane

### APPENDIX 2

### *Summarized data from IARC Monograph Series*

1. Rat and mouse negative

| Compound | Route (rat) | Route (mouse) | Reference |
|---|---|---|---|
| Aniline | p.o. | s.c. | 27 **4** (1974) |
| $\gamma$-Butyrolactone | p.o.<br>s.c. | p.o.<br>s.c. | 231 **11** (1976) |
| Cis-9, 10-Epoxystearic acid | s.c. | s.c.<br>top | 153 **11** (1976) |
| Maleic hydrazide | p.o.<br>s.c. | p.o.<br>s.c. | 173 **4** (1974) |
| Norgesterol | p.o. | p.o. | 201 **6** (1974) |
| Ponceau SX | p.o.<br>s.c. | p.o.<br>s.c. | 207 **8** (1975) |
| Yellow AB | p.o.<br>s.c. | <br>s.c. | 279 **8** (1975) |
| Yellow OB | p.o.<br>s.c. | <br>s.c. | 287 **8** (1975) |

2. Rat and mouse positive

| Compound | Rat: Route | Rat: Site affected | Mouse: Route | Mouse: Site affected | Reference |
|---|---|---|---|---|---|
| Amitrole | s.c., p.o. | Thyroid, liver | p.o. | Thyroid, liver | 31 **7** (1974) |
| o-Aminoazotoluene | p.o. | Liver, bladder | i.p., s.c., p.o. | Liver, lung | 61 **8** (1975) |
| 4-Aminobiphenyl | s.c. | Mammary gland, intestine | p.o. | Bladder, liver | 74 **1** (1972) |
| Aramite | p.o. | Liver | p.o. | In 1 of 4 strains tested: liver | 39 **5** (1974) |
| Asbestos | i.pl, i.h. | Lung | i.h. | Lung | 17 **2** (1972); 14 **2** (1977) |
| Benzidine | s.c., p.o. | Liver | s.c. | Liver | 80 **1** (1972) |
| Benzo(a)pyrene | top., i.t., i.v., p.o. | Mammary gland, forestomach | top., i.p., p.o. | Forestomach | 91 **3** (1975) |
| N,N-bis(2-chloroethyl)-2-naphthylamine | s.c. | Local tumours | i.p. | Lung | 119 **4** (1974) |
| Bis(chloromethyl)ether | s.c., i.h. | Lung, nasal cavities | s.c., top., i.h. | Lung | 231 **4** (1974) |
| β-Butyrolactone | s.c., p.o. | Tumours at site of admin. | top., s.c. | Tumours at site of admin. | 225 **11** (1976) |
| Cadmium salts | s.c. | Interstitial-cell tumours of testis | s.c. | Interstitial-cell tumours of testis | 74 **2** (1973) |
| Carbon tetrachloride | s.c., i.h. | Liver | p.o. | Liver | 53 **1** (1972) |
| Chlorambucil | i.p. | Lymphomas | i.p. | Ovary, lung | 125 **9** (1975) |
| Chromium salts Calcium chromate | i.b. | Lung | i.h. | Lung | 100 **2** (1973) |
| Citrus Red No. 2 | p.o. | Bladder | s.c., p.o. | Bladder | 101 **81** (1975) |
| Cycasin | p.o. | Liver, kidney | top., p.o. | Liver, lung, kidney | 157 **1** (1972) |
| Cyclophosphamide | s.c., i.p. | Lung, liver, reproductive organs | i.v., i.p. | Mammary gland | 135 **9** (1975) |
| Diazomethane | i.h. | Lung | s.c., i.h., top. | Lung | 223 **7** (1974) |
| Dibenz(a,h)anthracene | s.c., i.t. | Lung | top., p.o. | Forestomach | 178 **3** (1973) |
| 7H-Dibenz($C_{19}$) carbazole | s.c. | 1 expt: sarcomas, no details | top., s.c., p.o. | Forestomach, liver | 260 **3** (1973) |
| 1,2-Dibromo-3-chloropropane | p.o. | Forestomach, mammary gland | p.o. | Forestomach | 139 **15** (1977) |
| Diethylstilboestrol | s.c. | Mammary gland, pituitary | p.o., s.c. | Mammary gland, cervix, vagina (f), testis (m) | 55 **6** (1974) |
| Dihydrosafrole | p.o. | Oesophagus | p.o. | Liver (m), lung | 231 **10** (1976) |
| 1,2-Dimethylhydrazide | s.c., p.o. | Intestine, lung | s.c., p.o. | Liver, lung, muscle | 145 **4** (1974) |
| Ethinyloestradiol | p.o. | Liver | p.o. | Pituitary, mammary gland | 77 **6** (1974) |
| Ethylene dibromide | p.o. | Forestomach | p.o. | Forestomach | 195 **15** (1977) |
| Ethylmethane sulphonate | i.p. | Lung | s.c., i.p. | Lung, kidney | 245 **7** (1974) |
| Ethynodiol diacetate | p.o. | Mammary gland (benign) (m) | p.o. | Mammary gland (castrated) (m) | 173 **6** (1974) |
| 2[2-Formylhydrazino]-4-(5-nitro-2-furyl)thiazole | p.o. | Mammary gland, gastrointestinal tract | p.o. | Stomach, lung | 151 **7** (1974) |
| Isosafrole | p.o. | Liver | p.o. | Liver | 231 **10** (1976) |
| Melphalan | i.p. | Peritoneum | i.p. | Lung | 167 **9** (1975) |

| Compounds | Rat: Route | Rat: Affected organ | Mouse: Route | Mouse: Affected organ | Reference |
|---|---|---|---|---|---|
| Mestranol | p.o. | Mammary gland (f) | p.o. | Mammary gland, pituitary | 87 **6** (1974) |
| Methyl methanesulphonate | i.p., s.c. | Nervous system | i.p., p.o. | Lung; thymic lymphomas | 253 **7** (1974) |
| N-methyl-n-nitro-n-nitrosoguanidine | i.p., p.o. | Stomach, forestomach, liver | top., i.p., p.o. | Stomach | 183 **4** (1974) |
| Methylthiouracil | s.c., p.o. | Thyroid, kidney (f) | p.o. | Thyroid | 53 **7** (1974) |
| Metronidazole | p.o. | Mammary gland | p.o. | Lung | 113 **13** (1977) |
| Nickel salts | | | | | 75 **11** (1976) |
| Nickel subsulphide | i.h. | Lung | | | |
| Nickel subsulphide, nickel oxide | i.m. | Local tumours | i.m. | Local tumours | |
| 5-Nitroacenapthene | p.o. | Intestine, mammary gland (f) | i.p. | Leukaemia, reticulum cell sarcoma | 319 **16** (1978) |
| N-[4-(5-nitro-2-furyl)-2-thiazolyl] acetamide | p.o. | Mammary, salivary glands, lung, renal pelvis | p.o. | Forestomach | 185 **7** (1974) |
| Nitrogen mustard | i.v. | Variety of tumours | s.c., i.p., i.v. | Lung, thymus | 193 **9** (1975) |
| Nitrogen mustard n-oxide hydrochloride | i.v. | Lymphoreticular tumours | s.c. | Lung, thymus, Harderian gland | 209 **9** (1975) |
| N-nitrosodiethylamine | i.h., i.p., p.o. | Liver | top., i.p., s.c., p.o. | Liver, forestomach, oesophagus, lung | 107 **1** (1972) |
| N-nitrosodimethylamine | i.p., i.h., p.o. | Liver, kidney | s.c., i.p., p.o. | Liver, lung | 95 **1** (1972) |
| Nitrosoethylurea | s.c., i.v., p.o. | Brain, peripheral nervous system | i.p. | Multiple tumours, including intra-cranial, neurogenic | 135 **1** (1972) |
| Nitrosomethylurea | top., i.p., i.v., p.o. | Brain, peripheral nervous system | top., s.c., i.p. | Lung; thymic lymphomas | 125 **1** (1972) |
| Norethisterone | p.o. | Liver (m) (benign) | s.c., p.o. | Liver (m, benign) pituitary (f) | 179 **6** (1974) |
| Norethynodriel | p.o. | Liver, pituitary | s.c., p.o. | Pituitary | 191 **6** (1979) |
| Oestradiol 17$\beta$ | s.c. | Mammary gland, pituitary | s.c. | Mammary gland, pituitary, reproductive system | 99 **6** (1974) |
| Oestrone | s.c. | Pituitary, mammary gland | s.c., p.o. | Mammary gland | 123 **6** (1974) |
| Phenobarbitone | p.o. | Liver, benign | p.o. | Liver, benign/malignant | 157 **13** (1977) |
| Ponceau MX | p.o. | Liver | p.o. | Liver | 189 **9** (1975) |
| $\beta$-Propiolactone | p.o. | Forestomach | top., i.p. | Liver (m), lymphomas | 259 **4** (1974) |
| Propylthiouracil | p.o. | Thyroid | p.o. | Thyroid, pituitary | 67 **7** (1974) |
| Safrole | p.o. | Liver | p.o. | Liver | 231 **10** (1976) |
| Sterigmatocystin | top., p.o. | Liver | p.o. | Lung | 245 **10** (1976) |
| Streptozotocin | i.v., i.p. | Kidney, liver | i.p. | Lung, kidney | 337 **17** (1978) |
| Thiouracil | p.o. | Thyroid | p.o. | Liver | 85 **7** (1974) |
| Uracil mustard | i.p. | Peritoneum, pancreas, ovary, mammary gland | i.p. | Lung, liver, ovary | 235 **9** (1975) |
| Urethane | i.p., p.o. | Liver, uterus etc. | i.h., i.p., s.c. | Lung, liver etc. | 111 **7** (1974) |
| Vinyl chloride | i.h. | Liver, Zymbal gland, kidney | i.h. | Lung, mammary gland. liver | 291 **7** (1974) |

3. Rat results (all p.o.) negative, mouse positive

| Compound | Mouse Route | Reference |
|---|---|---|
| 1,4-Butanediol dimethane sulphonate | i.v.* | 247 **4** (1974) |
| DDT | p.o. | 83 **5** (1974) |
| Dieldrin | p.o. | 125 **5** (1974) |
| Isonicotinic acid hydrazide | s.c., i.p., p.o. | 159 **4** (1974) |
| 2-Naphthylamine | p.o. | 97 **4** (1974) |
| Trichlorethylene[1] | p.o. | 263 **11** (1976) |

[1] Trichloroethylene also included in NCI list. IARC opinion based on early report of NCI data.
* p.o. negative.

4. Rat results positive, mouse negative

| Compound | Rat Route | Mouse Route | Reference |
|---|---|---|---|
| Daunomycin | i.v. | p.o. | 145 **10** (1976) |
| p-Dimethylamino-azobenzene | top., i.p., p.o. | top. | 125 **8** (1975) |
| Thiourea | p.o. | p.o. | 95 **7** (1974) |
| Aflatoxin $B_1$ | i.p., p.o. | p.o. | 51 **10** (1976) |

5. Different results from various species

| Compound | Neg. Species | Neg. Route | Pos. Species | Pos. Route | Reference |
|---|---|---|---|---|---|
| Arsenic compounds | Rat, Mouse | p.o. | Man | top. | 48 **2** (1973) |
| Chlormadinone acetate | Rat, Mouse | p.o. | Dog | p.o. | 149 **6** (1974) |
| 3,3′-Dimethylbenzidine | Hamster | p.o. | Rat | s.c., i.p. | 87 **1** (1972) |
| Hydrazine | Hamster | p.o. | Rat, Mouse | p.o., i.p., p.o. | 127 **4** (1974) |
| Thioacetamide | Hamster | p.o. | Rat, Mouse | p.o. | 77 **7** (1974) |

Routes: In addition to the usual abbreviations are the following:
p.o.—gavage or in diet.
top.—topical.
i.h.—inhalation.
i.pl.—intrapleural injection.
i.b.—intrabronchial pellets.

### APPENDIX 3

### *Summarized carcinogenicity results from references derived from U.S. Public Health Service Publication No.* 149

1. Compounds negative in both rat (R) and mouse (M) (excluding compounds in Appendix 2)

| Compound | M/R | Route | Reference |
|---|---|---|---|
| (Acetato) phenylmercury | M | i.vag. | Boyland & Roe (1964) *Br. Emp. Cancer Campaign*, **42**, 22. |
| | R | p.o. | Fitzhugh *et al.* (1950) *AMA Arch. Ind. Hyg.*, **2**, 433. |
| Acetone | M | top. | Roe *et al.* (1970) *Br. J. Cancer*, **24**, 788. |
| | R | top.[2] | Glucksmann & Cherry (1968) *Br. J. Cancer*, **22**, 545. |
| Adipic acid dioctyl ester | M | top., s.c. | Hodge *et al.* (1966) *Tox. Appl. Pharmacol.*, **9**, 583. |
| | R | p.o. | |

| Compound | M/R | Route | Reference |
|---|---|---|---|
| Aniline (or aniline hydrochloride) | M | s.c.[2] | Hartwell & Andervont, *In* Hartwell & Shubick (1951) 50. |
| | R | p.o. | Druckrey (1950) *Arch. Exp. Path. Pharmakol.*, **210**, 137. |
| Anthracene | M | s.c. | Steiner (1955) *Cancer Res.*, **15**, 632. |
| | R | s.c.[2] | Schmahl (1955) *Krebsforsch.*, **60**, 697. |
| Arabinose | M | s.c. | Hueper (1965) *Cancer Res.*, **25**, 440. |
| | R | s.c. | |
| Arachis oil | M | s.c. | Boyland & Sims (1967) *Int. J. Cancer*, **2**, 500. |
| | | | Carter *et al.* (1969) *Fd. Cosmet. Tox.*, **7**, 53. |
| | R | s.c. | Dickens & Jones (1964) *Br. Emp. Cancer Campaign*, **42**, 141. |
| | | | Dickens & Jones (1965) *Br. J. Cancer*, **19**, 392. |
| Azobenzene | M | s.c.[2] | Shear & Stewart (1941) *In* Hartwell & Shubick (1951). |
| | R | s.c. | Spitz *et al.* (1950) *Cancer*, **3**, 789. |
| Benzene-1-azo-2-naphthol | M | p.o. | Clayson *et al.* (1965) *Br. J. Cancer*, **19**, 297. |
| | R | p.o.[2] | Hackmann (1951) *Krebsforsch.*, **57**, 530. |
| Benzene hexachloride ($\gamma$ isomer) | M | top. | Orr (1948) *Nature*, **162**, 189. |
| | R | p.o. | Fitzhugh *et al.* (1950) *J. Am. Pharm. Assoc.*, **40**, 583. |
| Benzoyl peroxide | M | p.o. | Sharrat *et al.* (1964) *Fd. Cosmet. Toxicol.*, **2**, 527. |
| | R | p.o. | |
| 2-Biphenylol | M | p.o. | Innes *et al.* (1969) *J. Natl Cancer Inst.*, **42**, 1101. |
| | R | p.o. | Hodge *et al.* (1952) *J. Pharmacol. Exp. Therap.*, **104**, 202. |
| 3,6-Bis(dimethylamino)acridine (Acridine Orange) | M | s.c. | Van Duuren *et al.* (1969) *Br. J. Cancer*, **23**, 587. |
| | R | s.c.[2] | |
| Camphor | M | i.p., top. | Stoner *et al.* (1973) *Cancer Res.*, **33**, 3069. |
| | | | Graffi *et al.* (1953) *Arch Geschwulstforsch*, **5**, 110. |
| | R | s.c. | Ezeyza (1952) *Semana Med.*, **100**, 663. |
| Carboxymethylcellulose | M | p.o. | McElligott & Hurst (1968) *Fd Cosmet. Toxicol.*, **6**, 449. |
| | R | p.o. | Teller *et al.* (1970) *Cancer Res.*, **30**, 179. |
| 2-Chloro-4,6-bis(ethylamino)-s-triazine (Simazin) | M | p.o. | Innes *et al.* (1969) *J. Natl Cancer Inst.*, **42**, 1101. |
| | R | s.c., p.o. | Pliss & Zabezhinsky (1970) *Vopr. Onkol.*, **16**, 81. |
| Cholesterol | M | s.c. | Bischoff (1957) *J. Natl Cancer Inst.*, **19**, 977. |
| | R | i.p. | Koch (1963) *Arzneimittelforschung*, **13**, 1116. |
| CI Acid Blue 9, diammonium salt (Brilliant Blue) | M | s.c. | Hansen *et al.* (1966) *Tox. Appl. Pharmacol.*, **8**, 29. |
| | R | p.o. | |
| CI Acid Green 5, disodium salt (Light Green SF Yellowish) | M | p.o. | Hansen *et al.* (1966) *Fd Cosmet. Toxicol.*, **4**, 389. |
| | R | p.o. | |
| CI Acid Red 26, isodium salt (Ponceaux MX) | M | p.o. | Waterman & Lignac (1958) *Acta Physiol Pharmacol. Neerl.*, **7**, 35. |
| | R | p.o. | Ikeda *et al.* (1966) *Fd Cosmet. Toxicol.*, **4**, 485. |
| CI Food Blue 1, disodium salt (FD & C Blue No. 2) | M | s.c. | Hansen *et al.* (1966) *Tox. Appl. Pharmacol.*, **8**, 29. |
| | R | p.o. | |
| CI Food Green 3, disodium salt (Fast Green FCF) | M | p.o. | Hansen *et al.* (1966) *Fd. Cosmet. Toxicol.*, **4**, 389. |
| | R | p.o. | |
| CI Food Red 1, disodium salt (Ponceaux SX) | M | p.o. | Davis *et al.* (1966) *Tox. Appl. Pharmacol.*, **8**, 306. |
| | R | p.o. | |
| CI Solvent Yellow 5 (phenylazo-2-naphthylamine) | M | s.c.[2] | Hansen *et al.* (1963) *Tox. Appl. Pharmacol.*, **5**, 16. |
| | R | s.c., p.o.[2] | |
| CI Solvent Yellow 6 (I-(2-methylphenyl)azo-2-naphthalenamine) | M | s.c. | |
| | R | s.c., p.o.[2] | |
| Cyclohexanesulfanic acid, monosodium salt | M | p.o. | Rudali *et al.* (1969) *C. R. Acad. Sci.*, **269**, 1910. |
| | R | p.o. | Grasso *et al.* (1971) *Fd Cosmet. Toxicol.*, **9**, 463. |
| Cyclohexene hydroperoxide | M | s.c. | Van Duuren *et al.* (1966) *J. Natl Cancer Inst.*, **37**, 825. |
| | R | s.c. | |
| D-glucose | M | s.c. | Hueper (1965) *Cancer Res.*, **25**, 440. |
| | R | s.c. | |
| 2,6-Dichloro-4-nitroaniline | M | p.o. | Innes *et al.* (1969) *J. Natl Cancer Inst.*, **42**, 1101. |
| | R | p.o. | Hadidian *et al.* (1968) *J. Natl Cancer Inst.*, **41**, 985. |
| Dicyclohexylamine | M | s.c. | Pliss (1958) *Vopr. Onkol.*, **4**, 659. |
| | R | p.o. | |
| 3,3′-Dihydroxybenzidine | M | s.c. | Bonser *et al.* (1956) *Br. J. Cancer*, **10**, 533. |
| | R | s.c. | Pliss (1961) *Vopr. Onkol.*, **7**, 33. |
| p-Diethylaminoazobenzene | M | s.c. | Kirby (1947) *Cancer Res.*, **7**, 333. |
| | R | s.c.[2] | |
| 17$\alpha$,21-Dihydroxypregn-4-ene-3,11,20-trione (Cortisone) | M | s.c. | Della Porta *et al.* (1970) *Tumori*, **56**, 121. |
| | R | p.o. | Field (1959) *Cancer Res.*, **19**, 870. |

| Compound | M/R | Route | Reference |
|---|---|---|---|
| a,a-Dimethylbenzyl hydroperoxide | M | s.c. | Van Duuren *et al.* (1966) *J. Natl Cancer Inst.*, **37**, 825. |
| | R | s.c.[2] | Van Duuren *et al.* (1967) *J. Natl Cancer Inst.*, **39**, 1213. |
| o,o-Dimethyl-1-hydroxy-2,2,2-trichlorethylphosphonate (Dipterex, Trichlorphon) | M | top. | Gibel *et al.* (1971) *Arch. Geschwulstforsch*, **37**, 303. |
| | R | s.c. | |
| 2-(2-(2-(dodecyloxy)ethoxy)ethoxy) ethanol | M | top. | Tusing *et al.* (1962) *Tox. Appl. Pharmacol.*, **4**, 402. |
| | R | p.o. | |
| 3-(Dodecyloxy)-1,2-propanediol-1-(hydrogen sulphate), sodium salt | M | top. | |
| | R | p.o. | |
| 9,10-Epoxystearic acid | M | s.c. | Van Duuren *et al.* (1966) *J. Natl Cancer Inst.*, **37**, 825. |
| | R | s.c. | |
| Ergosterol | M | top. | Barry *et al.* (1935) *Proc. R. Soc. Lond.* [*Biol.*], **117**, 318. |
| | R | i.p.[2] | Pizzolato & Beard (1945) *Exp. Med. Surg.*, **3**, 95. |
| Ethanol | M | p.o. | Kuratsune *et al.* (1971) *Gann*, **62**, 395. |
| | R | p.o.[2] | Yamamoto *et al.* (1967) *Int. J. Cancer*, **2**, 337. |
| (Ethylenebis(dithiocarbamato)) Manganese (Maneb) | M | p.o. | Innes *et al.* (1969) *J. Natl Cancer Inst.*, **42**, 1101. |
| | R | p.o. | Andrianova & Alekseev (1970) *Vopr. Pitan.*, **29**, 71. |
| (Ethylenebis(dithiocarbamato)) Zinc (Zineb) | M | p.o. | Innes *et al.* (1969) *J. Natl Cancer Inst.*, **42**, 1101. |
| | R | p.o. | Smith *et al.* (1953) *J. Pharmacol. Exp. Therap.*, **109**, 159. |
| Hexamethylenetetramine (Urotropin) | M | p.o. | Della Porta *et al.* (1968) *Fd Cosmet. Toxicol.*, **6**, 707. |
| | R | p.o.[2] | Della Porta *et al.* (1970) *Tumori*, **56**, 325. |
| 4-Hydroxy-3-nitrobenzenearsonic acid | M | p.o. | Prier *et al.* (1963) *Tox. Appl. Pharmacol.*, **5**, 526. |
| | R | p.o. | |
| Indole | M | s.c. | Felistovich (1964) *Vopr. Onkol.*, **10**, 70. |
| | R | p.o. | McDonald *et al.* (1962) *J. Urol.*, **87**, 381. |
| Isopropyl-N-(3-chlorophenyl) carbamate | M | p.o.[2] | Innes *et al.* (1969) *J. Natl Cancer Inst.*, **42**, 1101. |
| | R | p.o. | Larson *et al.* (1960) *Tox. Appl. Pharmacol.*, **2**, 659. |
| Lactose | M | s.c. | Hueper (1965) *Cancer Res.*, **25**, 440. |
| | R | s.c. | |
| Lauroyl peroxide | M | s.c. | Van Duuren *et al.* (1966) *J. Natl Cancer Inst.*, **37**, 825. |
| | R | s.c.[2] | Van Duuren *et al.* (1967) *J. Natl Cancer Inst.*, **39**, 1213. |
| Maltose | M | s.c. | Hueper (1965) *Cancer Res.*, **25**, 440. |
| | R | s.c. | |
| 1-Naphthyl-N-methylcarbamate (Crag Sevin) | M | p.o.[2] | Innes *et al.* (1969) *J. Natl Cancer Inst.*, **42**, 1101. |
| | R | p.o. | Andrianova & Alekseev (1970) *Vopr. Pitan.*, **29**, 71. |
| N-dodecylguanidine acetate | M | p.o. | Innes *et al.* (1969) *J. Natl Cancer Inst.*, **42**, 1101. |
| | R | p.o. | Levinskas *et al.* (1961) *Tox. Appl. Pharmacol.*, **3**, 127. |
| N,N-diphenylnitrosamine | M | p.o. | Innes *et al.* (1969) *J. Natl Cancer Inst.*, **42**, 1101. |
| | R | i.p. | Boyland *et al.* (1968) *Eur. J. Cancer*, **4**, 233. |
| Ochratoxin A | M | s.c.[2] | Dickens & Waynforth (1968) *Br. Emp. Cancer Campaign*, **46**, 108. |
| | R | s.c., p.o.[2] | Purchase & Van der Watt (1971) *Fd Cosmet. Toxicol.*, **9**, 681. |
| Piperonyl butoxide | M | p.o. | Innes *et al.* (1969) *J. Natl Cancer Inst.*, **42**, 1101. |
| | R | p.o. | Sarles & Vandegrift (1952) *J. Trop. Med. Hyg.*, **1**, 862. |
| Piperonyl ether butoxide | M | p.o.[2] | Innes *et al.* (1969) *J. Natl Cancer Inst.*, **42**, 1101. |
| | R | p.o.[2] | Sarles & Vandergrift (1952) *J. Trop. Med. Hyg.*, **1**, 862. |
| Polyethylene glycols | M | p.o. | Roe *et al.* (1970) *Fd. Cosmet. Toxicol.*, **8**, 135. |
| | R | i.p. | Boyland *et al.* (1968) *Eur. J. Cancer*, **4**, 233. |
| Polyvinyl pyridine-n-oxide | M | i.v. | Schmähl (1969) *Arzneimittelforschung*, **19**, 1313. |
| | R | i.v. | |
| Procaine penicillin | M | i.m. | Gilman & Ruckerbauer (1962) *Cancer Res.*, **22**, 152. |
| | R | i.m. | |
| 1,2-Propanediol (propylene glycol) | M | top. | Fujino *et al.* (1965) *J. Natl Cancer Inst.*, **35**, 907. |
| | R | s.c. | Hine *et al.* (1958) *Arch. Ind. Hlth*, **17**, 129. |
| Sorbose | M | s.c. | Hueper (1965) *Cancer Res.*, **25**, 440. |
| | R | s.c. | |
| Sucrose | M | s.c. | |
| | R | s.c. | |
| Sulfosuccinic acid, 1,4-bis-(2-ethylhexyl)ester, sodium salt | M | p.o. | Klein (1963) *Cancer Res.*, **23**, 1701. |
| | R | p.o. | Fitzhugh & Nelson (1948) *J. Pharmacol. Exp. Therap.*, **93**, 147. |

| Compound | M/R | Route | Reference |
|---|---|---|---|
| Tricaprylin | M | s.c. | Van Duuren *et al.* (1966) *J. Natl Cancer Inst.*, **37**, 825. |
| | R | s.c. | |
| 1,1,1-Trichloro-2,2-bis(p-methoxyphenyl)ethane (methoxychlor) | M | top. | Hodge *et al.* (1966) *Tox. Appl. Pharmacol.*, **9**, 583. |
| | R | p.o. | Deichmann *et al.* (1967) *Tox. Appl. Pharmacol.*, **11**, 88. |

2. Compounds positive in both rat and mouse

| Compound | M/R | Route | Affected organ | Reference |
|---|---|---|---|---|
| 2-Acetylaminofluorene | M | p.o. | Liver, bladder | Wood (1969) *Eur. J. Cancer*, **5**, 41. |
| | R | p.o. | Liver, mammary gland | Peraino *et al.* (1971) *Cancer Res.*, **31**, 1506. |
| 2-Amino-2,5-azotoluene | M | p.o. | Liver | Crabtree (1948) *Br. J. Cancer*, **3**, 387. |
| | R | p.o. | Liver | |
| 2-Amino-fluorene | M | top. | Liver | Bielschowsky & Bielschowsky (1960) *Br. J. Cancer*, **14**, 195. |
| | R | top. | Liver | Goodall (1965) *Endocrinology*, **76**, 1027. |
| 2-Anthramine | M | top. | Mammary gland | Lennox (1955) *Br. J. Cancer*, **9**, 631. |
| | R | p.o. | Skin | Griswold *et al.* (1968) *Cancer Res.*, **28**, 924. |
| Bis(acetato)dihydroxytri-lead | M | p.o. | Kidney | Van Esch & Kroes (1969) *Br. J. Cancer*, **23**, 765. |
| | R | p.o. | Kidney, brain | Oyaswi *et al.* (1970) *Cancer Res.*, **30**, 1249. |
| Carbon tetrachloride | M | p.o. | Liver | Unakar (1966) *Arch. Path.*, **82**, 170. |
| | R | s.c. | Liver | Reuber & Glover (1970) *J. Natl Cancer Inst.*, **44**, 419. |
| 2,7-Diacetylaminofluorene | M | p.o. | Liver | Takayama (1968) *J. Natl Cancer Inst.*, **40**, 629. |
| | R | p.o. | Mammary gland, liver, intestine | Yamada *et al.* (1971) *Gann.*, **62**, 471. |
| p-Dimethylaminobenzene-1-azo-2-naphthalene | M | top. | Skin | Mulay & Saxen (1952) *J. Natl Cancer Inst.*, **13**, 1259. |
| | R | p.o. | Liver | Mulay & Longdon (1953) *J. Natl Cancer Inst.*, **14**, 571. |
| 7,12-Dimethylbenz(a)-anthracene | M | i.v. | Leukaemia, ovary | Uematsu & Higgins (1969) *Gann.*, **60**, 545. |
| | R | i.v. | Mammary gland | Geyer *et al.* (1953) *Cancer Res.*, **13**, 503. |
| 4′-Fluoro-4-aminodiphenyl | M | p.o. | Liver | Clayson *et al.* (1965) *Br. J. Cancer*, **19**, 297. |
| | R | s.c. | Kidney, liver | Matthews & Walpole (1958) *Br. J. Cancer*, **12**, 234. |
| Imuran | M | i.m. | Thymus etc. | Casey (1968) *Blood*, **31**, 396. |
| | R | p.o. | Zymbal gland | Frankel *et al.* (1970) *Tox. Appl. Pharmacol.*, **17**, 462. |
| 3-Methylcholanthrene | M | top., s.c. | Leukaemia, local | Rubin (1971) *Progr. Exp. Tumor Res.*, **14**, 138. Matsuyama *et al.* (1963) *Nature*, **197**, 805. |
| | R | p.o., s.c. | Mammary gland, local | Gruenstein *et al.* (1966) *J. Natl Cancer Inst.*, **36**, 483. Matsuyama *et al.* (1963) *Nature*, **197**, 805. |
| 7-Methylbenz(a)-anthracene | M | s.c. | Local | Miller & Miller (1963) *Cancer Res.*, **23**, 229. |
| | R | s.c. | Local | Pataki & Huggins (1969) *Cancer Res.*, **29**, 506. |
| N-Fluoren-2-yl acetohydroxamic acid | M | p.o. | Mammary gland, stomach, liver | Miller *et al.* (1964) *Cancer Res.*, **24**, 2018. |
| | R | p.o. | Liver, bladder | Weisburger *et al.* (1970) *J. Natl Cancer Inst.*, **45**, 29. |
| N-Isopropyl-a-(2-methyl- | M | i.p. | Leukaemia, lung | Kelly *et al.* (1969) *J. Natl Cancer Inst.*, **42**, 337. |
| hydrazino)-p-toluamide monochloride | R | i.p. | Mammary gland | Kelly *et al.* (1968) *J. Natl Cancer Inst.*, **40**, 1027. |
| N-nitrosobutylethylamine | M | p.o. | Stomach | Schmahl *et al.* (1963) *Naturwissenschaften*, **50**, 717. |
| | R | p.o. | Oesophagus | Thomas & So (1969) *Arzneimittelforschung*, **19**, 1077. |
| N-nitrosobutylurea | M | p.o. | Thymus, leukaemia | Yokoro *et al.* (1970) *Gann*, **61**, 287. |
| | R | p.o. | Zymbal gland | Odashima (1970) *Gann.*, **61**, 245. |

| Compound | M/R | Route | Affected organ | Reference |
|---|---|---|---|---|
| N-nitrosomethylaniline | M | p.o. | Lung, lymphoreticular | Greenblatt *et al.* (1971) *J. Natl Cancer Inst.*, **46**, 1029. |
| | R | p.o. | Lymphoreticular, stomach | Goodall *et al.* (1970) *Tox. Appl. Pharmacol.*, **17**, 426. |
| N-4-((5-Nitro-2-furyl)-2-thiazolyl)formamide | M | p.o. | Bladder | Erturk *et al.* (1970) *Cancer Res.*, **30**, 1309. |
| | R | p.o. | Bladder | Erturk *et al.* (1969) *Proc. Am. Assoc. Cancer Res.*, **10**, 23. |
| 3-Nitro-3-hexene | M | inh. | Lung | Deichmann *et al.* (1965) *Indus. Med. Surg.*, **34**, 800. |
| | R | inh. | Lung | |
| 4-Nitroquinoline 1-oxide | M | s.c. | Lung | Mori *et al.* (1966) *Gann.*, **57**, 559. |
| | R | s.c. | Lung | |
| 4-Nitrosopiperazine | M | p.o. | Lung | Greenblatt *et al.* (1971) *J. Natl Cancer Inst.*, **46**, 1029. |
| | R | p.o. | Lymphoreticular | Garcia *et al.* (1970) *Z. Krebsforsch*, **74**, 179. |
| 19-Nor-17α-pregn-1,3,5(10)-trien-20-yne-3,17-diol | M | p.o. | Mammary, uterine | Committee on Safety of Medicines (1972) Carcinogenicity tests of oral contraceptives, London; HMSO. |
| | R | p.o. | Mammary, liver | |

3. Rat results negative; mouse positive

| Compound | M/R | Route | Reference |
|---|---|---|---|
| 6-Aminochrysene | M | top. | Lambelin *et al.* (1975) *Eur. J. Cancer*, **11**, 327. |
| | R | p.o.[2] | Higgins (1964) *Proc. Natl Acad. Sci.*, **51**, 737. |
| 1,1-Dimethylhydrazine | M | p.o. | Toth (1972) *Proc. Am. Assoc. Cancer Res.*, **13**, 34. |
| | R | p.o. | Argus & Hoch-Ligeti (1961) *J. Natl Cancer Inst.*, **27**, 695. |

4. Rat results positive; mouse negative

| Compound | M/R | Route | Reference |
|---|---|---|---|
| 4-Aminostilbene | M | p.o. | Clayson *et al.* (1965) *Br. J. Cancer*, **19**, 297. |
| | R | p.o.[2] | Anderson *et al.* (1964) *Cancer Res.*, **24**, 128. |
| Oestrone | M | p.o. | Biancifiori *et al.* (1967) *Br. J. Cancer*, **21**, 452. |
| | R | s.c. | Cutts (1964) *Cancer Res.*, **24**, 1124. |
| Poly(1,2-dihydro-2,2,4-trimethyl-quinoline) | M | s.c., top. | Hodge *et al.* (1966) *Tox. Appl. Pharmacol.*, **9**, 583. |
| | R | p.o. | |
| Polyethyleneglycol monostearate | M | p.o. | Hueper & Payne (1963) *Arch. Env. Hlth*, **6**, 484. |
| | R | p.o. | |
| 4-Styrylacetanilide | M | p.o. | Clayson *et al.* (1965) *Br. J. Cancer*, **19**, 297. |
| | R | p.o. | Baldwin *et al.* (1968) *Br. J. Cancer*, **22**, 133. |

Footnote

[1] Data not quoted by IARC Monograph Vol. 4, p. 137.
[2] Animal group sizes relatively small.

Routes abbreviated as follows:
p.o.—gavage or addition to diet.
top.—topical application.
i.vag.—intravaginal instillation.